# Endoscopic treatment versus open excision for pediatric pilonidal disease: technical description of a modified (P)EPSiT approach using standard equipment and retrospective cohort study

**DOI:** 10.1007/s10151-026-03317-5

**Published:** 2026-05-04

**Authors:** J. Kirsch, S. Drossard, K. Schriek, U. Hübner

**Affiliations:** 1 Wilhelmstift Children’s Hospital, Liliencronstrasse 130, 22149 Hamburg, Germany; 2 Department of General, Visceral, Transplant, Vascular and Pediatric Surgery, University Hospital Würzburg, Oberdürrbacher Str. 6, 97080 Würzburg, Germany; 3 Auf der Bult Children’s and Youth Hospital, Janusz-Korczak-Allee 12, 30173 Hannover, Germany

**Keywords:** Pilonidal sinus, Pilonidal cyst, Endoscopy, Minimally invasive surgery, Pit picking, Adolescents

## Abstract

**Background:**

Endoscopic pilonidal sinus treatment (EPSiT) has emerged as a minimally invasive treatment option for pilonidal disease (PD) in adolescents, yet its adoption has been limited by the need for specialized equipment. We developed a modified EPSiT technique using standard urological instruments, saline irrigation, and lateral positioning, which was introduced in 2019. This study provides a technical description of the modified approach and evaluates the outcomes compared with conventional open excision in a pediatric cohort.

**Methods:**

We conducted a single-center retrospective cohort study of 113 pediatric patients treated surgically for PD between 2014 and 2023. Patients were divided into two cohorts: EPSiT (*n* = 48, 2019–2023) and open excision (*n* = 65, 2014–2018). Clinical data were collected from medical records for both groups and structured telephone interviews for patients undergoing EPSiT. Outcomes included operative time, hospital stay, recurrence, pain, and satisfaction.

**Results:**

Gender distribution was identical in both cohorts (29.2% male, 70.8% female). Operative times were similar between groups (35.8 versus 31.7 min; *p* = .307). EPSiT was associated with significantly shorter hospital stays (mean difference −2.55 days; 95% CI −3.10 to −2.00; *p* < .001). Recurrence rates were comparable (16.7% versus 15.4%; *p* = .808). Patient-reported outcomes were available for the EPSiT cohort only and indicated high cosmetic satisfaction and minimal analgesic use. Among patients undergoing EPSiT, 41.7% returned to school immediately after discharge, and most resumed normal activities within a few days.

**Conclusions:**

This modified EPSiT approach is feasible and may increase accessibility in resource-limited settings, representing a less invasive treatment option for PD. Further prospective studies are needed to validate these findings and define the role of EPSiT in the treatment of pediatric PD.

**Supplementary Information:**

The online version contains supplementary material available at 10.1007/s10151-026-03317-5.

## Introduction

Pilonidal disease (PD) is characterized by the presence of epithelialized sinus tracts containing hair fragments. Although PD was historically considered a congenital disorder, it is now widely accepted as an acquired condition caused by hair penetration and subcutaneous embedding, triggering a foreign body reaction and subsequent fistula formation [1]. PD primarily affects adolescents and young adults, with a higher incidence in males [2–4]. Risk factors include obesity, prolonged sitting, and local trauma [2], contributing to increasing incidence worldwide [5]. Patients with a family history of PD tend to develop the condition at a younger age (mean 21.5 ± 2.6 years) [6].

Traditionally, wide excision with open wound healing has been the standard surgical approach for PD and is the method of choice in many pediatric surgical facilities in Germany. Although this method is effective, it often results in prolonged wound healing and significant morbidity [7]. Various surgical techniques have been developed to improve outcomes, including the Limberg and Karydakis flaps. While these techniques have demonstrated lower recurrence rates, they are associated with high rates of wound dehiscence, impaired healing [7] and prominent scars. Minimally invasive techniques have gained popularity as alternatives: originally introduced by Meinero in 2014 [8], endoscopic pilonidal sinus treatment (EPSiT) is a minimally invasive technique that allows direct visualization and precise debridement of sinus tracts using an endoscopic system, aiming to preserve surrounding tissue and minimize postoperative morbidity. EPSiT has demonstrated faster healing, reduced postoperative pain, and superior cosmetic outcomes in adults [9–12]. It can be applied in both chronic PD and acute abscessing presentations [13].

More recently, the technique has been adapted for pediatric patients as pediatric endoscopic pilonidal sinus treatment (PEPSiT) [14, 15]. While initial studies report favorable outcomes regarding safety, effectiveness, and accelerated recovery [4, 15–19], existing literature on the efficacy of PEPSiT is scarce and primarily consists of small, retrospective case series with short follow-up durations. A recent systematic review of nine studies including 320 patients supports the safety and efficacy of PEPSiT and suggests potential advantages over conventional surgical approaches [20]. However, its adoption in pediatric surgical practice remains limited.

## Objective of the study

At our institution, EPSiT has been adapted for pediatric patients with a modified technique using a standard cystoscope. This manuscript provides a technical description and reports retrospective data comparing the clinical outcomes of EPSiT and open excision in children and adolescents, focusing on operative time, hospital stay, recurrence rates, postoperative pain, and patient satisfaction.

## Materials and methods

This retrospective study was conducted at Wilhelmstift Catholic Children’s Hospital (Hamburg, Germany), analyzing pediatric patients treated for PD between 2014 and 2023. Patients were divided into two cohorts: those treated with EPSiT (2019–2023) and those who underwent open excision (2014–2018), with or without vacuum therapy. All pediatric patients (0–17 years) treated surgically during the study period were included, except those with acne inversa or severe syndromic disabilities (e.g., Trisomy 13).

For both groups, data were extracted from medical records collecting epidemiological (age, gender, BMI) and perioperative data (operative time, hospital stay, antibiotic use, and intraoperative hair volume, categorized as few, moderate, or extensive).

Recurrence was defined as any postoperative presentation with clinical signs or symptoms compatible with PD (e.g., recurrent drainage, pain, swelling, abscess formation, or new pits) requiring medical or surgical treatment at our institution or reported by the patient/family as having been treated elsewhere during follow-up.

In addition, postoperative follow-up for group 1 was conducted through structured telephone interviews using a standardized questionnaire (Table 1). Patients were contacted and asked about recurrence rates, healing time, scar satisfaction, postoperative pain management, and time to return to school.Adherence to recurrence prevention measures (daily gluteal hygiene and hair removal) was also assessed.

**Table 1:** Interview guide (translated)

	Question	Categories
1	How long did you remain under medical treatment?	A few days, up to 1 week, up to 2 weeks, up to 4 weeks
2	How long did it take for the wound to close completely?	up to 1 week, up to 2 weeks, longer than 2 weeks
3	How long did you take pain medication?	At the hospital only, 1 day after discharge, not at all
4	Did you take antibiotics after discharge?	Yes/No
5	Did you require reoperation at the same site?	Yes/No
6	Were you treated at another hospital for this condition?	Yes/No
7	When did you return to school?	Immediately after discharge, within a few days, after 1 week
8	Did you follow a recurrence prevention protocol?	Yes/No
9	How satisfied are you with your scar?	Somewhat satisfied, very satisfied

Responses were documented verbatim and subsequently grouped into descriptive categories by the researcher using qualitative content clustering. The resulting categories are presented in Table 1.

Structured telephone follow-up was conducted for the EPSiT cohort only. Comparable patient-reported outcomes were not collected routinely during the earlier open-excision period; therefore, subjective outcomes are reported descriptively for patients undergoing EPSiT without formal between-group comparison.

## Data analysis

Primary outcomes were length of hospital stay and recurrence. Secondary outcomes included operative time and perioperative antibiotic use. Patient-reported outcomes (pain, satisfaction, return to school) were collected for the EPSiT cohort only and are reported descriptively. Data analysis was performed using SPSS Version 29 (SPSS Inc., Chicago, IL, USA). Continuous variables were expressed as means with 95% confidence intervals (CIs). Normality was assessed visually and with the Shapiro–Wilk test. Between-group comparisons for continuous data were performed using independent-samples *t*-tests when appropriate. Categorical variables were summarized as counts and percentages, and group comparisons were conducted using chi-square or Fisher’s exact tests. Associations between continuous variables were assessed with Pearson or Spearman correlation coefficients, as appropriate. *p*-values < 0.05 were considered statistically significant. Absolute differences and 95% CIs are reported for key comparisons. No adjustments were made for multiple comparisons; secondary analyses are considered exploratory. Associations between operative variables were assessed as exploratory analyses. We did not perform multivariable adjustment; therefore, all between-group comparisons should be interpreted as descriptive.

## Surgical protocol

From 2019 onward, all patients undergoing surgical management for pilonidal disease at the authors’ institution were treated using the modified EPSiT approach. The technique was applied to both acute abscessing presentations (including closed abscesses) and chronic disease, with or without ongoing secretion. Since its introduction, the procedure has been consistently performed under the supervision of two senior pediatric surgeons, who were present for all cases.

A full material list, step-by-step description of the procedure and “pearls and pitfalls” can be found in Supplementary File 1. A video depicting the procedure performed in a case of acute PD with abscess formation is available as Supplementary File 2.

**Fig. 1:**
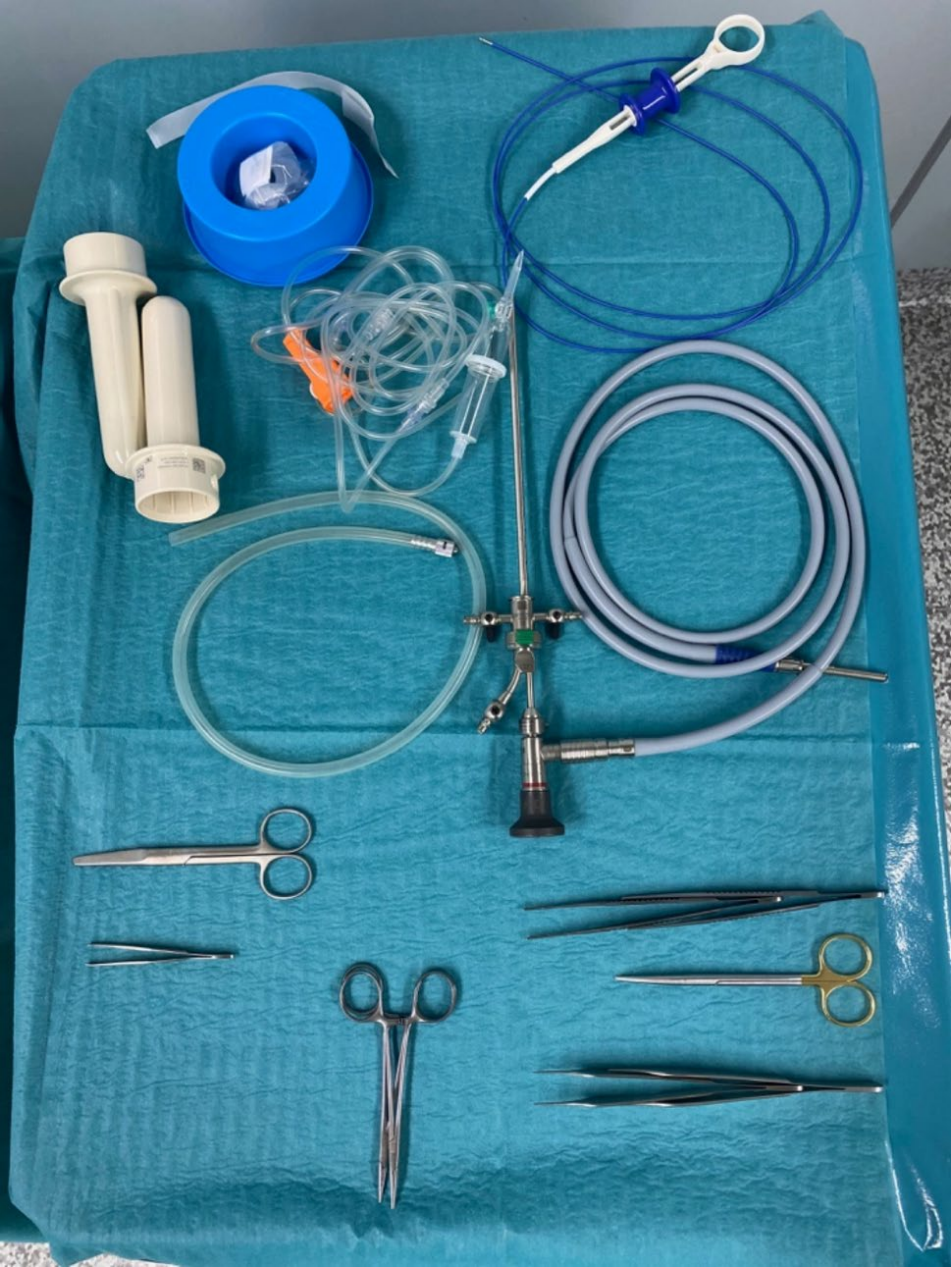
Material needed for the modified EPSiT procedure

Our modified approach employs a standard 12.5 Ch cystoscope with camera optics and a working channel, eliminating the need for dedicated fistuloscopes and making the procedure more accessible. Additionally, 0.9% sodium chloride irrigation replaces glycine-mannitol solution, providing a simpler, cost-effective alternative without compromising visualization, as described by Esposito et al. [14]. Figure 1 shows the material needed for the procedure.

**Fig. 2:**
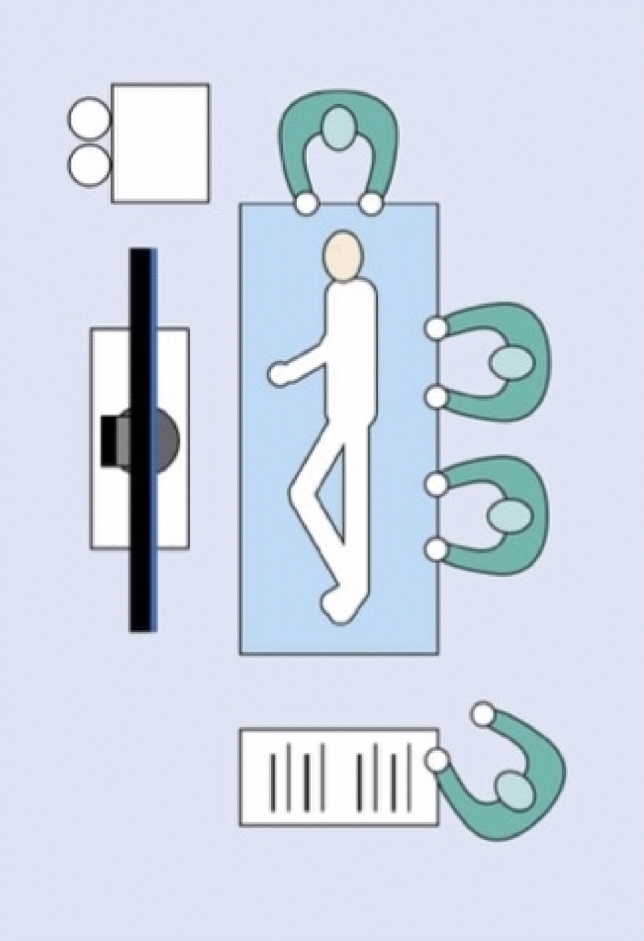
OR setup and patient position, schematic

**Fig. 3:**
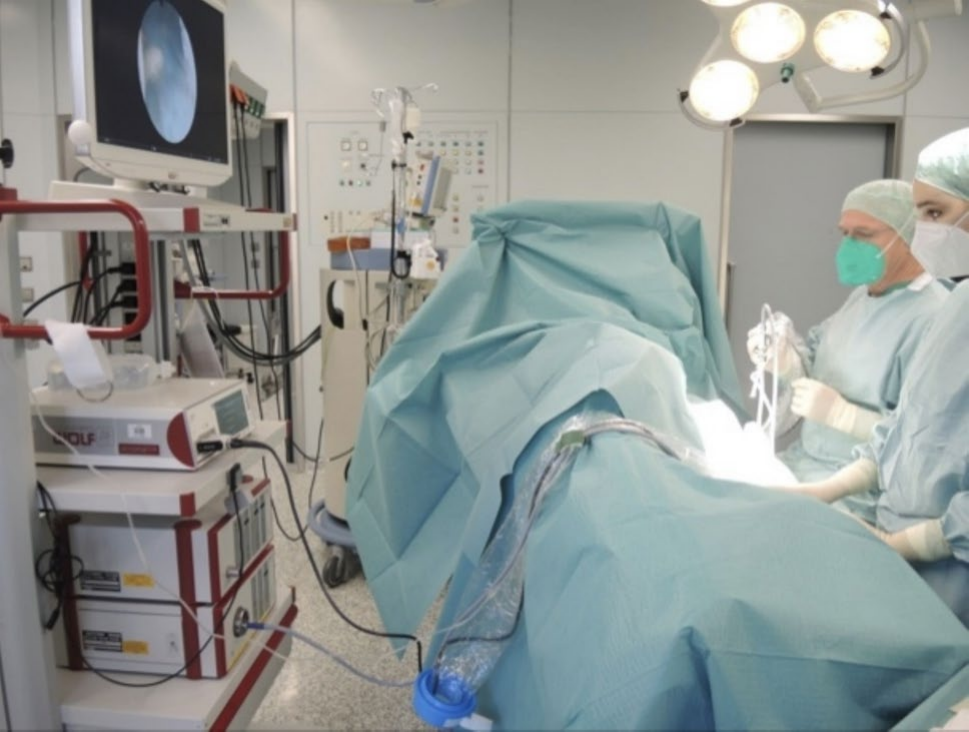
OR setup and patient positioning

Patients are placed under general anesthesia and positioned in the lateral decubitus position, which in our experience provides a practical alternative to the traditional prone position. This approach allows faster positioning and facilitates both ventilation and surgical access, particularly in obese patients. The surgeon and assistant operate from the patient’s dorsal side, with the monitor placed opposite the operating table to ensure optimal ergonomics with the scrub nurse at the patient’s feet (Figs. 2 and 3). The cystoscope is then introduced parallel to the operating table via existing pits, a stab incision, or a skin punch opening in cases of closed abscesses.

**Fig. 4:**
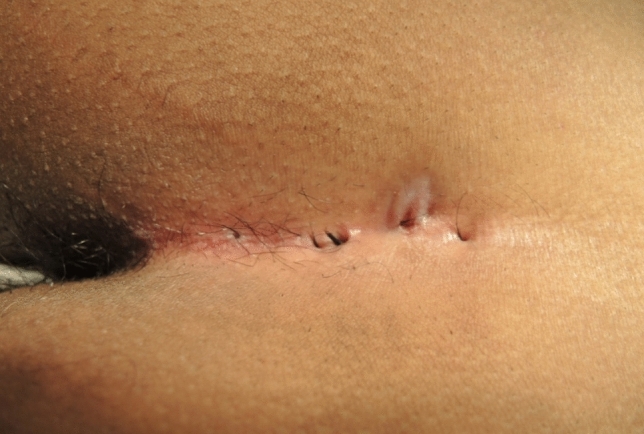
Pilonidal sinus disease without abscess, with hairs lying in the pits

**Fig. 5:**
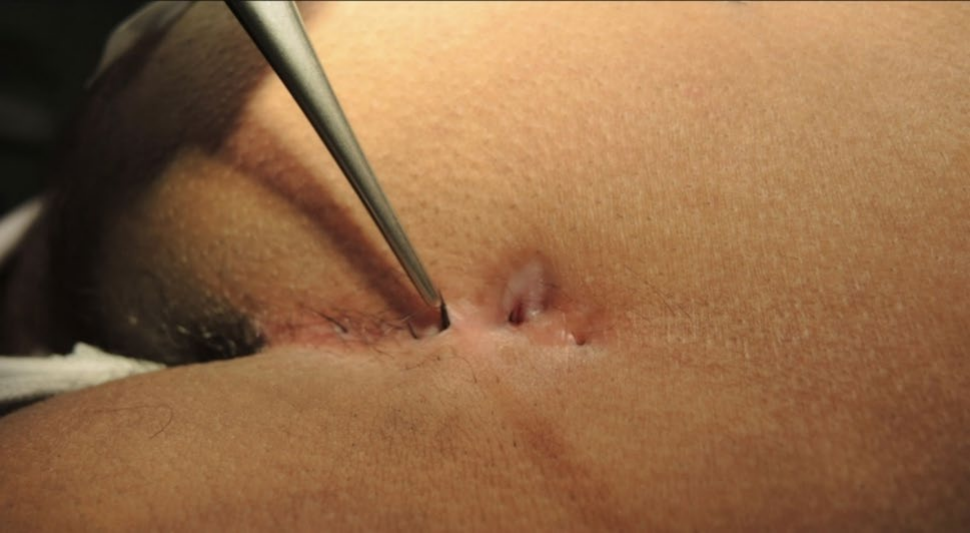
Visible hairs are removed with tweezers

**Fig. 6:**
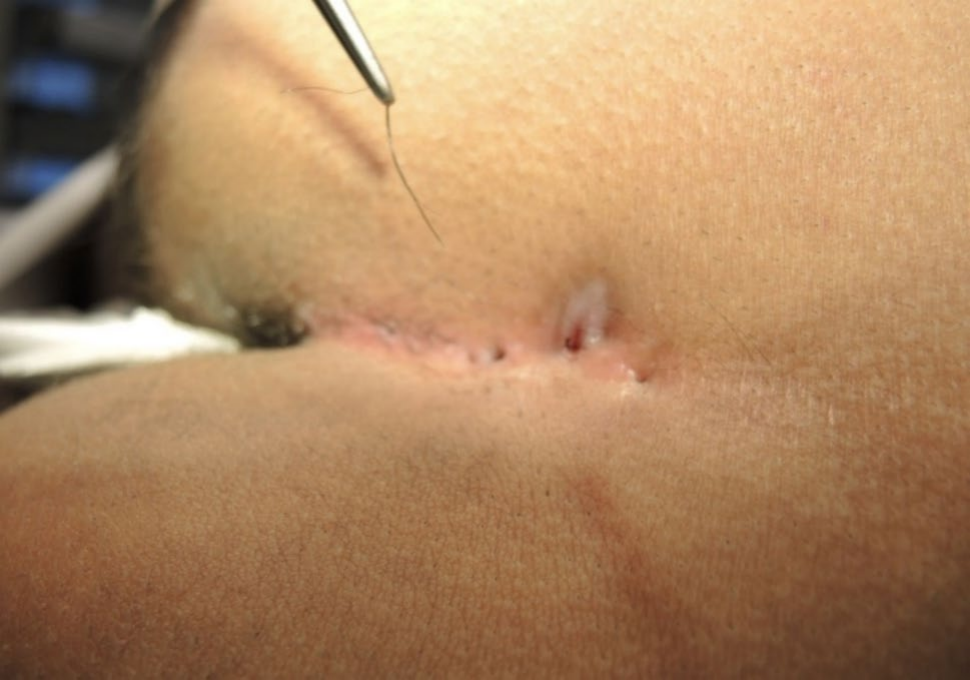
Hair removal with tweezers. Additionally, the sinus cavity is scraped out with a sharp spoon

**Fig. 7:**
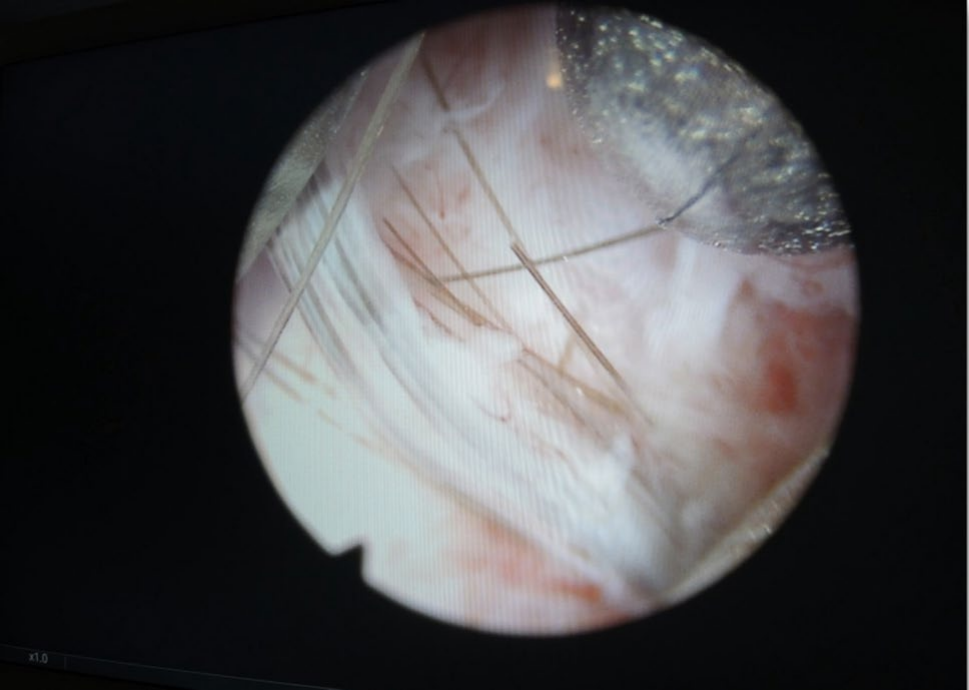
The view through the cystoscope into the sinus cavity

**Fig. 8:**
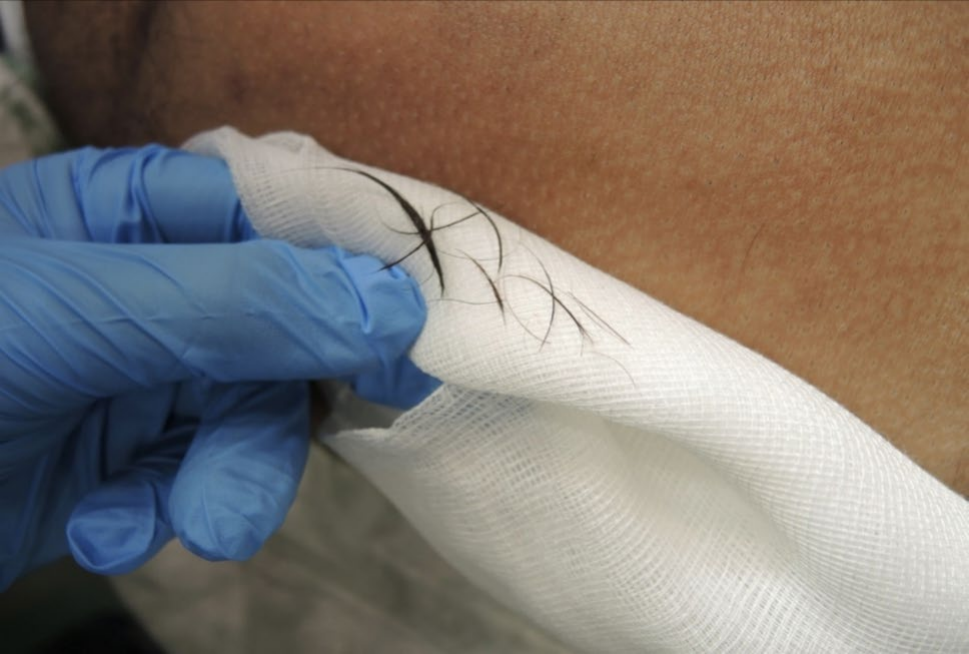
Hairs that could be retrieved endoscopically

Initially, complete endoscopic removal of all hairs within the sinus tract was attempted. However, it became evident that a combined approach of endoscopic inspection and pit picking was both sufficient and more efficient: The procedure begins with conventional pit picking and curettage. Ingrown hairs are extracted using forceps (Figs. 4, 5, and 6), and the sinus cavity is thoroughly debrided with a sharp spoon, followed by endoscopic inspection of the sinus tract (Fig. 7). If residual hairs are identified, they are excised either through repeat curettage or retrieved using endoscopic grasping forceps (Fig. 8). Endoscopic reinspection is performed after each debridement to ensure complete removal of hair and debris. This iterative process was found to simplify the procedure while maintaining surgical efficacy.

**Fig. 9:**
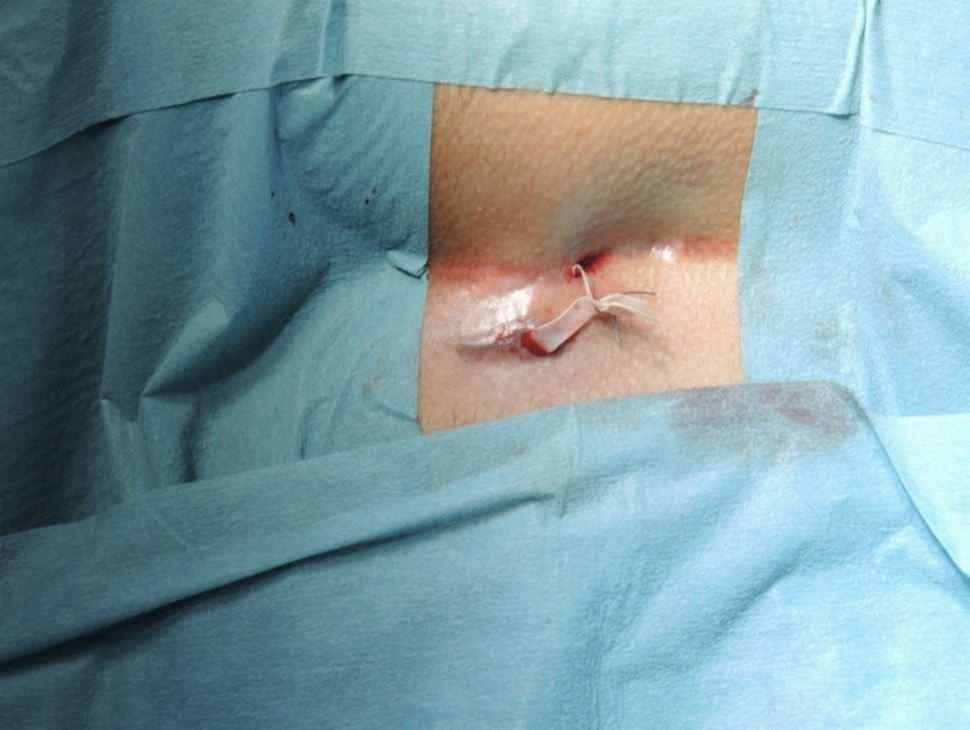
End of the procedure with loop in place

Noncommunicating sinus tracts are treated separately. In case of abscess formation, a counter incision is made in healthy tissue to ensure adequate drainage (Fig. 9).

All pits are routinely excised with a skin punch to remove epithelialized tissue and promote proper healing by secondary intention.

Patients in the earlier cohort underwent conventional excision under general anesthesia, with some receiving vacuum therapy for wound management. Depending on intraoperative findings, wound management was performed either by secondary intention, primary closure, or adjunctive negative-pressure wound therapy according to surgeon preference at the time.

## Results

A total of 113 pediatric patients underwent surgical treatment for PD and were divided into two cohorts: 48 patients received EPSiT (2019–2023), and 65 patients underwent open excision (2014–2018), some with adjunctive vacuum therapy. Baseline demographics were similar between groups (Table 2): both cohorts had a 29.2% male and 70.8% female distribution. Mean age was 14.60 years (95% CI 14.06–15.14) in the EPSiT group and 14.46 years (95% CI 14.02–14.91) in the open excision group. No statistically significant differences in age distribution were observed between male and female patients in either group (*p* = 0.724 for group 1; *p* = 0.9665 for group 2, *t*-test). Acute abscess at index surgery was present in 23/48 patients undergoing EPSiT (47.9%).

**Table 2:** Cohort description

Demographics	Group 1: EPSiT (2019–2023)	Group 2: open excision (2014–2018)
Total patients	48	65
Male	14 (29.2%)	19 (29.2%)
Female	34 (70.8%)	46 (70.8%)
Age (years)		
Mean	14.60	14.46
SD	1.55	1.44
Range	10–17	11–17

Mean operative time was 35.8 min (95% CI 28.78–42.84) in the EPSiT group and 31.7 min (95% CI 28.27–35.17) in the open excision group. The mean difference of 4.1 min (95% CI −3.75 to 11.93; *p* = 0.307) was not statistically significant. A negative correlation was found between the incision-suture time and the time since the introduction of EPSiT, though this was not statistically significant (*p* = 0.167, Fisher’s exact test). In the EPSiT cohort, operative time correlated with intraoperative hair volume (*r* = 0.381; *p* = 0.018), while no correlation was observed with hospital stay duration (*r* = 0.071; *p* = 0.639). In the open excision group, operative time correlated modestly with hospital stay (*r* = 0.257; *p* = 0.040). Longer operative times in male patients (mean 47.9 min) compared with female patients (mean 30.2 min) in the EPSiT group were not statistically significant (*p* = 0.175).

Hospital stay was significantly shorter in the EPSiT group (mean 2.43 days, SD 1.06, 95% CI 2.12–2.74) than in the open excision group (mean 4.98 days, SD 1.81, 95% CI 4.53–5.43), with a mean difference of −2.55 days (95% CI −3.10 to −2.00; *p* < 0.001) (Table 4). There was no significant association with gender (*p* = 0.561) or BMI (*p* = 0.329). Mean length of stay (LOS) after open excision remained relatively stable across 2014–2018 while LOS after EPSiT decreased over time from 3.75 days in 2019 to 1.75 days in 2023 (*r* =  −0.465; *p* < 0.001, Table 3).

**Table 3:** Length of stay and operative time by year and technique

Year	Technique	*n*	LOS in days, mean (SD)	OP time in min, mean (SD)
2014	Open Excision	11	5.00 (1.18)	28.5 (11.1)
2015		12	4.80 (1.62)	22.5 (7.5)
2016		13	4.46 (0.88)	32.5 (10.6)
2017		13	5.69 (2.39)	35.3 (17.0)
2018		16	5.25 (2.14)	37.1 (16.1)
2019	EPSiT	4	3.75 (2.22)	31.0 (14.4)
2020		7	2.43 (0.98)	43.6 (31.8)
2021		19	2.68 (0.82)	34.2 (23.0)
2022		10	1.89 (0.33)	37.6 (30.8)
2023		8	1.75 (0.71)	32.6 (16.1)

Perioperative antibiotics were administered in 92% (46/48) of EPSiT and 95% (62/65) of patients undergoing open excision, most commonly cefuroxime. Use of single-shot prophylaxis increased in the EPSiT group over time, reaching 39% by 2022. No significant association between antibiotic use and recurrence was observed (*p* = 0.123, Fisher–Freeman–Halton exact test).

Among patients undergoing EPSiT, 66.7% (32/48) reported sick leave duration: 41.7% (20/48) returned to school immediately after discharge, 14.6% (7/48) within a few days, and 10.4% (5/48) after 1 week. Most patients reported wound healing within 2 weeks, with only two minor complications attributed to residual hair or overlooked fistulas. Patient-reported satisfaction with the cosmetic result was uniformly high. Analgesic use was rare, with only 4.1% (2/48) taking antiinflammatory drugs during hospitalization. Data on wound healing and patient-reported outcomes were not available for the open excision group. Follow-up data from 12 patients in that group indicated a mean wound care duration of 11.2 months, with four requiring home nursing.

Recurrence was reported in 16.7% (8/48) of patients undergoing EPSiT and 15.4% (10/65) in the open excision group (Table 4). The absolute difference of 1.28% (95% CI −12.43 to 15.00%; *p* = 0.808, chi-square test) was not statistically significant. All patients with recurrence had received perioperative antibiotics. Among patients undergoing EPSiT, two early recurrences occurred within 6 weeks and were characterized by persistent secretion from pits. These were considered suggestive of residual disease or incomplete tract clearance, while later events (> 1.5 years) may reflect new sinus formation. Recurrence was not significantly associated with gender (*p* = 0.776), BMI (*p* = 0.267), intraoperative hair volume (*p* = 0.102), or operative time (*p* = 0.597). Recurrence occurred in 3/23 patients (13.0%) with an acute abscess and 5/25 (20.0%) without an abscess. This difference was not statistically significant (*p* = 0.70, Fisher’s exact test).

**Table 4:** Comparison of outcome

Outcome	Group 1: EPSiT (2019–2023), *n* = 48			Group 2: Open Excision (2014–2018), *n* = 65			*p* value
	Mean	SD	Range	Mean	SD	Range	
Procedural duration (min)	35.81	24.22	13–119	31.72	13.91	10–78	0.307
Hospital stay (days)	2.42	1.06	1–7	4.98	1.81	2–11	< 0.001
Recurrence rate (%)	8 (16.7%)			10 (15.4%)			0.808
	Absolute difference: 1.28% (95% CI −12.43% to 15.00%)						

In the open excision group, no significant correlations were observed with gender (*p* = 0.413) or operative time (*p* = 0.222). BMI could not be analyzed due to missing data in this cohort. Despite medical recommendations, no patients in group 1 adhered to recurrence prevention measures, such as daily gluteal hygiene or hair removal.

## Discussion

Our study supports EPSiT as a viable alternative to open excision in pediatric PD, with significantly shorter hospital stays, quick return to daily activities, and high patient satisfaction in our cohort.

A recent systematic review confirmed the safety and efficacy of EPSiT for pediatric patients [20]; however, variations in equipment and technique remain across institutions. Our study contributes to this discussion by evaluating a modified EPSiT approach that eliminates the need for specialized endoscopic equipment, making it more accessible in surgical centers without dedicated fistuloscopes.

Our results align with previous studies demonstrating favorable outcomes with EPSiT [4, 16, 20–22]. Compared with 5–7 days reported for flap procedures and open excision in a German sample [23], our results indicate that EPSiT is associated with shorter hospitalization. Additionally, 41.7% of patients were able to return to school immediately postdischarge, and the majority resumed normal activities within a few days. Although literature suggests EPSiT can be performed as an outpatient procedure [18], the longer inpatient stays in our cohort were likely owing to the learning curve. As surgical experience increased, discharge times progressively shortened, reflecting greater confidence in postoperative management and patient recovery. As the procedure became more reliable, postoperative antibiotic use shifted from multi-day regimens to single-shot prophylaxis, with no impact on recurrence rates. These time trends likely reflect evolving perioperative pathways and increasing familiarity with EPSiT during implementation.

Although PD severity scores have been proposed [21], no universally accepted grading system exists, limiting comparability between cohorts because baseline disease extent may differ. In our study, formal severity staging was not feasible because a validated score was not routinely used and key variables were incompletely documented retrospectively. Adjunctive vacuum therapy in a subset of open excision cases may reflect more severe presentations.

The recurrence rate in our EPSiT group (16.7%) aligns with previously published data in pediatric populations [16, 20]. However, recurrence definitions vary widely in the literature, complicating direct comparison. Factors such as missed or incompletely excised fistulas, impaired healing, and new fistula formation likely contribute to the range of reported recurrence rates [24]. None of our patients adhered to recommended recurrence prevention strategies, such as regular hair removal or daily gluteal cleansing, despite these being potentially beneficial in reducing recurrence risk [25, 26]. This may reflect limited health literacy and low adherence among adolescents, as well as financial barriers, as laser therapy is not covered by German health insurance. Improved patient education, age-appropriate counseling, written instructions, and structured follow-up should be implemented to increase adherence to postoperative care recommendations.

In our cohort, 70.8% of patients were female. This female predominance is consistent with Oetzmann et al., who observed a higher prevalence of pilonidal disease in girls during early adolescence [5]. However, larger contemporary pediatric series generally report an overall male predominance [3, 4]. The discrepancy may reflect local referral patterns and the age distribution of patients treated at our institution.

Patient-reported outcomes were collected only for the EPSiT cohort, limiting direct comparison of pain, satisfaction, and time to return to normal activities between techniques. These findings should therefore be interpreted as descriptive observations of the EPSiT experience rather than comparative evidence. A major advantage of EPSiT is minimal postoperative pain. Most patients in our cohort did not require analgesics beyond the immediate postoperative period, consistent with reports of pain scores approaching zero in an Italian study [10]. In contrast, open excision and flap procedures often result in prolonged pain and discomfort due to larger wound surfaces and high-tension closure techniques [27]. Wound healing outcomes were favorable, with high patient and parental satisfaction. The small, puncture-like scars left by EPSiT were perceived as cosmetically excellent, a particularly important factor for adolescents. This aligns with previous studies, where 85% of patients reported high levels of scar satisfaction [11]. For adolescent patients, especially girls, the cosmetic outcomes are of greater concern than in the typically older, predominantly male adult population. Given the psychological impact of large surgical scars in this age group, EPSiT provides a significant aesthetic advantage over total excision and flap-based techniques in pediatric patients.

Notably, EPSiT was performed as a single-stage procedure in 23 patients presenting with acute abscessing PD, thereby avoiding a staged approach with delayed definitive surgery. Recurrence was numerically lower in patients operated during an acute abscess compared with nonabscess cases; however, this difference was not statistically significant, and the underlying reasons remain unclear. Future prospective studies should evaluate whether abscess presentation at surgery influences outcomes after endoscopic treatment.

While the 2020 German S3 guideline does not recommend endoscopic approaches because of limited evidence and continues to prioritize off-midline flap (OMF) techniques (such as the Limberg and Karydakis flaps), an updated version is currently under revision [26]. In contrast, a 2025 international Delphi consensus reported that most participating experts favored EPSiT as the preferred option for both primary and recurrent PD in adults (90%) and children (80%) [28]. Comparative data on EPSiT and OMF procedures remain limited, with only two studies directly comparing these techniques: While OMF procedures may be associated with lower recurrence rates, they require longer operative times and carry a higher risk of complications. In contrast, EPSiT offers a minimally invasive approach with fewer postoperative complications, but it may be associated with a higher recurrence risk [22, 29]. In the pediatric population, a single-center retrospective study evaluating patients aged 1–21 years found that OMF reconstruction had lower recurrence and reoperation rates than EPSiT, but it was associated with longer operative times and higher wound complication rates [22]. Similarly, a prospective, nonrandomized study comparing EPSiT with the Limberg flap procedure in adults with complicated PD demonstrated a lower success rate for EPSiT but also fewer complications [29]. These findings highlight the importance of individualized surgical decision-making for PD in all patients. An online survey found that patients may be willing to accept trade-offs between shorter recovery times and a higher risk of complications such as infection or persistent disease [30]. Therefore, as Romaniszyn et al. [29] suggest, patients and families should be counseled on the risks and benefits of each approach, offering them a choice between EPSiT—a minimally invasive procedure with a higher recurrence risk—and OMF, a more effective alternative associated with longer recovery time and higher complication rate.

## Limitations

This study offers comparative data on EPSiT and open excision in pediatric patients. However, several limitations must be acknowledged. The retrospective study design limits control over confounding variables, and the absence of long-term follow-up prevents an assessment of recurrence rates beyond the study period. Additionally, the reliance on self-reported results and lack of a standardized recurrence definition makes direct comparisons with other studies challenging. Future prospective studies should incorporate standardized severity documentation as well as validated patient-reported outcome measures to quantify pain, quality of life, and cosmetic satisfaction. Furthermore, this study compares EPSiT to open excision only and does not include comparisons with off-midline closure techniques such as the Limberg or Karydakis flap procedures. Future research should include comparative analyses with flap-based procedures, providing a more comprehensive evaluation of surgical options and their respective outcomes in pediatric patients.

A key limitation is temporal confounding: the cohorts represent sequential time periods, and institutional pathways evolved over the study decade. In addition, increasing surgeon experience with EPSiT may have contributed to shorter hospitalization and changes in antibiotic use. Consequently, observed differences cannot be attributed to surgical technique alone, and causal inference is limited. These limitations emphasize the exploratory nature of our findings, which serve to inform future studies. Prospective, multicenter, randomized studies are needed to validate these findings and further establish EPSiT as a standard approach for pediatric PD treatment.

## Conclusions

EPSiT is a safe and well-tolerated alternative to open excision for pediatric PD and is associated with short hospital stays, fast recovery, minimal pain, and satisfactory cosmetic outcomes. While recurrence rates are comparable to conventional techniques, adherence to recurrence prevention strategies may further improve long-term outcomes.

Our approach utilizes a standard cystoscope, proving that EPSiT can be performed without specialized equipment, making it a cost-effective option, particularly for resource-limited healthcare settings. Given these advantages, EPSiT is now the standard approach at our institution. We suggest considering it as a treatment option for patients seeking minimally invasive surgery with rapid recovery. However, owing to relevant recurrence rates, surgical decision-making should involve a balanced discussion of EPSiT versus off-midline flap procedures, ensuring an individualized approach tailored to patient preferences.

## Supplementary Information

Below is the link to the electronic supplementary material.

Supplementary file1 (DOCX 20 KB)

Supplementary file2 (MOV 1307382 KB)

## Data Availability

The datasets generated during and/or analysed during the current study are available from the corresponding author on reasonable request.
