# Supplementary material for: Endoscopic treatment versus open excision for pediatric pilonidal disease: technical description of a modified (P)EPSiT approach using standard equipment and retrospective cohort study
Source: Tech Coloproctol. 2026 May 4;30(1):59. doi: 10.1007/s10151-026-03317-5 (PMC13139232; doi:10.1007/s10151-026-03317-5)
Supplement: Supplementary file 1 — Supplementary file1 (DOCX 20 KB) [file 10151_2026_3317_MOESM1_ESM.docx]

**Supplement 1: Step-by-Step Technical Description for modified EPSiT**

| Indication |
| --- |
| Acute PD presenting with an abscess (including closed abscess).  Chronic PD with midline pits/sinus openings, with or without persistent secretion.  Recurrent PD after previous surgical treatment |
| Material List |
| Endoscopic equipment   - Rigid cystoscope, 12.5 Ch, with camera optics and working channel (incl. light source and monitor) - Irrigation tubing/stopcock system compatible with the cystoscope - 0.9% sodium chloride irrigation bags (with giving set / pressure bag if used)   Instruments   - Endoscopic grasping-forceps (must fit through the working channel) - Dermal punch, 3 mm (for pit excision/pit punching) or 11-blade scalpel (for stab incision) - Sharp curettes (small and medium) or sharp spoon - Anatomical forceps/tweezers (various sizes)   Wound Care   - Sterile gauze swabs (10 × 10 cm) - Dressing materials   Drainage (in abscessing cases)   - Irrigation solution (e.g. NaCl 0,9% or polyhexanide) and syringe for irrigation - Vessel loop or small silicone drain - Scissors |
| Preparation and Setup |
| - General Anesthesia (or Spinal Anesthesia if feasible) - Single Shot Antibiotic Therapy - Place the patient in right lateral decubitus position. - Surgeon and assistant stand dorsally. The monitor is positioned opposite the table. The scrub nurse stands at the patient’s feet. - Prep and drape the intergluteal region widely. |
| Surgical Steps |
| 1. Access creation   - Identify all midline pits and any secondary openings. - Use an existing pit as the primary entry. If needed, enlarge a pit with the punch to permit atraumatic scope entry and to excise epithelialized pit edges. - If pits are closed in acute abscessing PD, create access by 3-mm dermal punch over the point of maximal fluctuance.   2. Initial debridement   - Express purulent/serous discharge if present. - Remove visible hairs and loose debris with tweezers or by curettage. (Figure 5) - Using a small sharp curette, curette the pit and superficial cavity to remove debris and reduce turbidity before endoscopy.   3. Endoscopic exploration and irrigation   - Introduce the 12.5 Ch cystoscope parallel to the operating table. - Irrigate continuously with 0.9% saline. - Systematically inspect the cavity and identify all communicating tracts and side branches.   4. Hair removal and Cavity debridement   - Perform thorough mechanical debridement of granulation tissue and epithelial remnants using the curette (reintroduce as needed). - If single hairs persist, extract them through the working channel using endoscopic grasping forceps.   🡪 Under endoscopic control, Repeat Steps 2-4 (debridement followed by endoscopic inspection) until no residual hair or debris is seen and the cavity walls appear clean.  5. Pit Excision and Wound management   - Excise epithelialized pit edges using the 3-mm punch to facilitate secondary healing and reduce persistence of epithelialized openings. - Leave wounds to heal by secondary intention and apply an absorbent dressing.   Abscess-specific steps   - Perform a counter-incision in healthy tissue for adequate drainage. - Place a vessel loop or small silicone drain. |
| Pearls and Pitfalls |
| - If needed retract the buttocks with tape to open the cleft. - Instead of a skin punch, a simple stab incision can be made. This may need to be widened using forceps to fit the endoscope. - Thoroughly debride the cavity with a curette before introducing the endoscope. This step facilitates later visualization and is repeated as needed. - Hair extraction via the working channel is useful for single strands but is inefficient as the primary method: Use endoscopy to identify side tracts and to confirm clearance after curettage rather than trying to extract every hair endoscopically. - Non-communicating cavities require separate access and complete debridement for each: If non-communicating cavities are present, repeat steps 1-4 for each cavity separately, establishing additional punch access as required. - Increase irrigation pressure temporarily if visualization is poor (debris/bleeding). - Refreshing of pit edges helps prevent persistence of epithelialized openings. |
